# Adolescent morphine exposure does not alter low-dose lipopolysaccharide (LPS)-induced sickness behavior in adult C57/BL6 mice

**DOI:** 10.1371/journal.pone.0328026

**Published:** 2025-11-04

**Authors:** Natalie V. Davidson, Cynthia Masese, Caitlin Han, Lili Massac, Yuu Ishikawa, Grace Reynolds, Codey Smithson, Jackson Cook, Melissa T. Manners, Shivon A. Robinson

**Affiliations:** 1 Department of Psychology, Williams College, Williamstown, Massachusetts, United States of America; 2 Department of Biological and Biomedical Sciences, Rowan University, Glassboro, New Jersey, United States of America; PLOS: Public Library of Science, UNITED STATES OF AMERICA

## Abstract

Adolescent opioid use in the United States commands attention: millions of twelve- to nineteen-year-olds are exposed to opioids each year by prescription and misuse. Recent findings show that opioids bind not only to canonical opioid receptors but also interact with receptors on immune cells within both the central and peripheral nervous systems. The potential for early life opioid exposure to give rise to long-term changes in the neuroimmune system is not fully understood, particularly given the adolescent brain’s high susceptibility to neuroplastic changes. The goal of this study was to investigate the hypothesis that adolescent opioid use potentiates physiological and behavioral responses to lipopolysaccharide (LPS)-induced sickness later in life. To achieve this, we treated adolescent (postnatal day 35–42) male and female C57/BL6 mice with saline or bi-daily escalating doses of morphine for 5 days to model opioid dependence and, in adulthood (postnatal day 60–67), administered saline or a low dose of LPS (0.1 mg/kg) to promote an immune response. Body weight, body surface temperature, and locomotor activity were recorded up to 48 hours after LPS administration. Mice were also tested in the forced swim test 52 hours after LPS administration to assess depressive-like behavior. In contrast to our hypotheses, we found that adolescent morphine exposure had no additive effect on low-dose LPS-induced sickness measures when assessed in adulthood. These data suggest that adolescent opioid exposure may have minimal effects on future immune challenges, although further research is needed to confirm this.

## Introduction

Despite the declining rate of dispensed opioid prescriptions [1] and opioid-related overdoses [2], opioid misuse and addiction remain critical public health issues in the United States. A growing concern within the current opioid crisis is the rising cases of adolescent opioid use. In 2023, approximately 5.7 million Americans aged 12 and older had an opioid use disorder, and 8.6 million reported misusing prescription painkillers [3]. Notably, a multi-center retrospective study discovered that among 12–17 year-olds, the incidence of opioid-related presentations to the emergency department increased 2800% between 2014 and 2022 [4]. Nonmedical use of prescription opioids during adolescence is correlated with high-risk behaviors, such as violence victimization, poor academic performance, and suicidal thoughts [5]. Furthermore, adolescent opioid use is associated with increased risk of developing opioid dependence and substance use disorder symptoms later in life [6,7]. Because adolescence is a critical neurodevelopmental period in which individuals may be more susceptible to the effects of psychoactive substances on brain functioning [8–10], there is a pressing need to further elucidate the neurobiological impact of adolescent opioid exposure on long-term health outcomes.

Opioids classically exert their analgesic and euphoric effects via binding to endogenous opioid receptors located throughout the central and peripheral nervous system [11–13]. Opioids can also modulate immune signaling through interactions with opioid receptors expressed on various immune cells, including lymphocytes and macrophages [14–16]. Prior work has indicated that opioids have immunosuppressive effects in the periphery [17–20]. However, opioids can also bind to the accessory proteins of the Toll-Like Receptor 4 (TLR4) [21,22], which is canonically activated by pathogen-associated endotoxins, such as lipopolysaccharide (LPS), and stimulates the production of proinflammatory cytokines [23,24]. Opioid-mediated activation of TLR4 receptors within the central nervous system (CNS) has been shown to lead to neuroinflammation [22,25,26].

Elevated pro-inflammatory cytokine signaling in the CNS triggers complex neuroimmune mechanisms that manifest as distinct physiological and behavioral responses, often referred to as “sickness behaviors” [27,28]. In rodents, LPS-induced sickness is characterized by decreased motor activity, weight loss, and body temperature changes, in addition to increased depressive-like behaviors [29–32], though many of these effects are dose- and time-dependent. Sickness behaviors can be adaptive and promote recovery by shifting energy allocation toward fighting infection [33]. However, prolonged or excessive immune activation may have detrimental outcomes. For example, chronic neuroinflammation is associated with the development of depression [34–36] and has been proposed as a key factor in modulating the impacts of social-environmental adversity and stress on overall health and lifespan [37].

Chronic opioid exposure can dysregulate immune system functioning, potentially leading to increased susceptibility to infection and maladaptive immune responses [14,38–40]. Notably, administration of a mu-opioid receptor antagonist reduces LPS-induced inflammation and sickness behavior in mice [41–43]. Although the majority of this work has been conducted in adult models, there is growing evidence indicating that early-life opioid exposure is disruptive to future immune functioning. In humans, prenatal opioid exposure is associated with higher expression levels of inflammatory genes [44] and increased risk of childhood infection [45]. Additionally, in rodent models, perinatal opioid exposure stimulates immune hyperactivity in the brain and periphery [46,47] and exacerbates LPS-induced neuroinflammatory responses and sickness behaviors in adulthood [48,49].

Adolescence is also a vulnerable developmental period for immune insults [50–52]. In rats, adolescent morphine exposure potentiates morphine-induced neuroimmune activation and drug-seeking behavior in adulthood [53]. However, to the best of our knowledge, there has been no preclinical investigation into whether adolescent opioid exposure specifically alters LPS-induced sickness behaviors later in life. Moreover, although opioid exposure in adult mice can produce sexually dimorphic neuroimmune responses [54], it is unclear how sex differences manifest in the context of an adult immune challenge following adolescent opioid exposure. To investigate this, we administered morphine to male and female adolescent mice using a 5-day escalating dose paradigm. In adulthood, the mice were exposed to a low dose of LPS (0.1 mg/kg) and assessed for physiological and behavioral signs of sickness, including alterations in body weight, body surface temperature, and locomotion. To determine whether adolescent morphine exposure alters LPS-induced depressive-like behavior in adulthood, mice were also assessed in the forced swim test (FST), a widely used measure of behavioral despair. We hypothesized that adult mice with a history of adolescent morphine exposure would exhibit more severe LPS-induced sickness symptoms compared to saline controls.

## Materials and methods

### Animals

Male and female C57BL/6NTac mice (n = 65) obtained from Taconic Biosciences were used for all experiments. Mice were maintained on a 12-hour dark-light cycle (lights on at 07:00) in a temperature and humidity-controlled facility with food and water available ad libitum. All experiments were performed in compliance with the National Institutes of Health’s Guide for Care and Use of Laboratory Animals, and protocols were approved by the Institutional Animal Care and Use Committee at Williams College (Protocol #: RS-A-22). Mice were carefully handled and monitored throughout the study to minimize discomfort and stress. Once experiments were completed, mice were sacrificed using carbon dioxide (CO_2_) asphyxiation followed by decapitation, per established guidelines for humane euthanasia of rodents. Using the “simr” package in R [55], we conducted a simulation-based power analysis on preliminary data examining the effects of LPS on locomotor activity. A sample size of 6 mice per group was estimated to provide 80% power with a 95% confidence interval.

### Drug treatment

Morphine sulfate was obtained from Millipore Sigma (St. Louis, MO) and dissolved in 0.9% sterile saline. Adolescent mice (postnatal day 35–42) received repeated subcutaneous injections of morphine beginning on the evening of Day 1 and then twice daily (at 9 AM and 4 PM) at escalating doses (10, 20, 30, 40, or 50 mg/kg), with the final injection occurring on the morning of Day 5. An escalating dosing schedule has previously been shown to induce dependence in adolescent animals [56]. Controls received saline at identical time points. Mice were weighed daily during morphine treatment and up to 1 week after the final injection. Following adolescent morphine exposure, mice were left undisturbed until adulthood.

**Fig 1:**
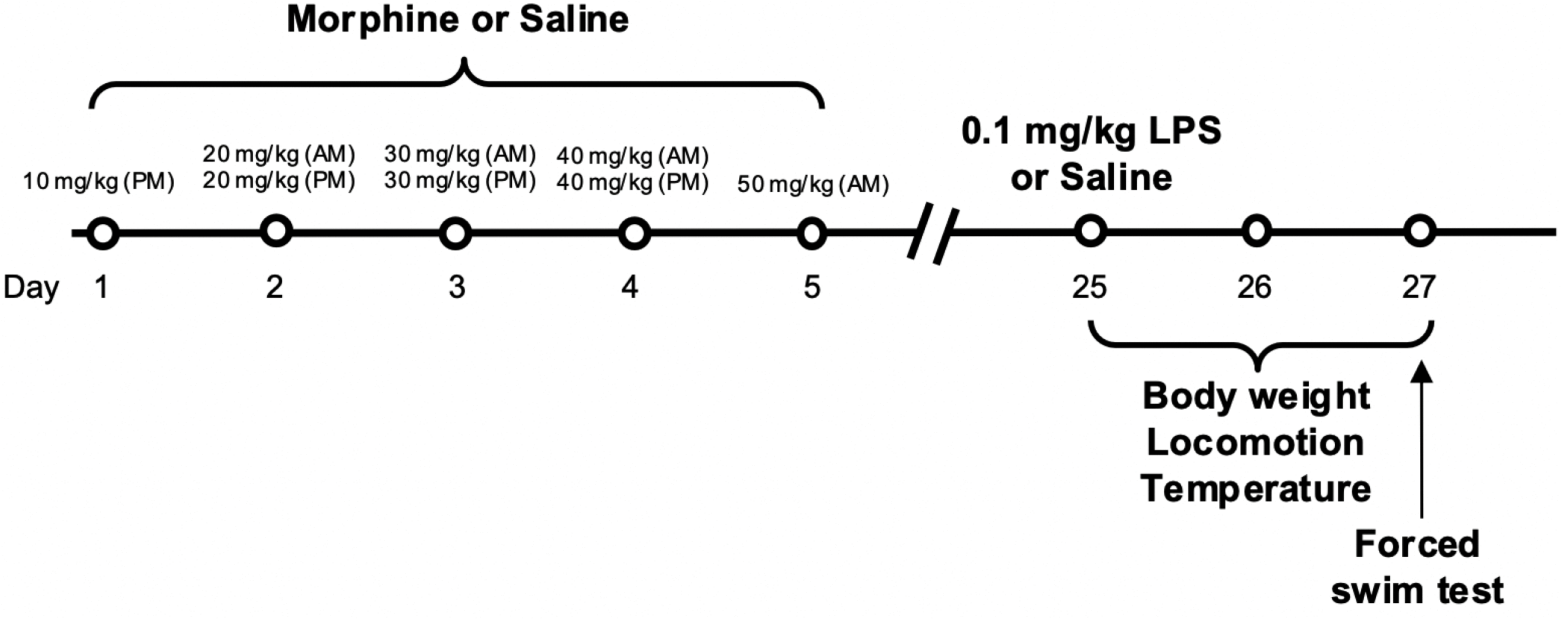
Experimental timeline.

Lipopolysaccharide (LPS) derived from *E. coli* 0111: B4 was obtained from Millipore Sigma (St. Louis, MO) and dissolved in 0.9% sterile saline. During adulthood (postnatal day 60–67), mice were administered a single intraperitoneal injection of LPS (0.1 mg/kg) or saline. This dose was chosen due to its ability to elevate pro-inflammatory cytokine levels and generate mild to moderate sickness symptoms [32,57] while having minimal effects on affective behaviors in the absence of an additional insult [58]. Following LPS administration, mice were assessed for several physiological and behavioral signs of sickness. An overview of the experimental timeline is shown in Fig 1.

All drugs were administered in an injection volume of 10 ml/kg, and controls received sterile saline at an equal volume.

### Physiological measures

Mice were weighed immediately before the LPS or saline injection, and then 24 hours and 48 hours following the injection. Body surface temperature was measured via an infrared thermometer (iProven), which prior work has demonstrated to be a reliable measure of body temperature in mice [59,60]. The thermometer was aimed at the lower abdomen immediately before LPS or saline administration, and then 1, 24, and 48 hours after administration.

### Locomotor activity

The effect of LPS on locomotor activity was assessed 1, 24, and 48 hours after administration. After 30 minutes of acclimation to the experimental room, mice were then individually placed in a 20 x 20 x 35 cm acrylic apparatus for 30 minutes. Distance traveled in meters was measured using ANY-maze video tracking software (Stoelting Co., Wood Dale, IL).

### Forced swim test

The forced swim test (FST) was conducted to investigate the effect of adolescent morphine exposure on adult LPS-induced depressive-like behavior. Mice were tested 52 hours after LPS administration. This time point was selected based on preliminary data indicating that the locomotor-suppressing effects of LPS dissipated by 48 hours post-injection. After 30 minutes of acclimation to the experimental room, mice were then placed individually in a plastic cylinder (26 cm tall x 18 cm diameter) filled with water (24 °C + /- 1 °C) to a depth of 15 cm for six minutes. Total time immobile in the last 4 minutes of the 6-minute test was measured using Anymaze tracking software (Stoelting Co., Wood Dale, IL). A period of immobility was defined as 2 seconds or greater of no movement [61].

### Statistical analysis

Data were analyzed and graphed using the package “afex” within R [62] and GraphPad Prism 9 software, respectively. Data were analyzed using linear models including sex, morphine treatment, LPS treatment, and the interaction term as fixed effects. Linear mixed-effects models were used for datasets that included repeated measurements, and also included time point as a fixed effect and subject as a random effect. Statistical outliers were identified using the two-sided Grubbs’ test (α = 0.05) and excluded from analysis. Post-hoc analyses were performed using Bonferroni’s post-hoc tests. Statistical significance was set at p < 0.05 throughout. Statistical tables for all statistical models can be found in the supplemental information (S1–S5 Tables).

## Results

### Adolescent morphine exposure reduces body weight

**Fig 2:**
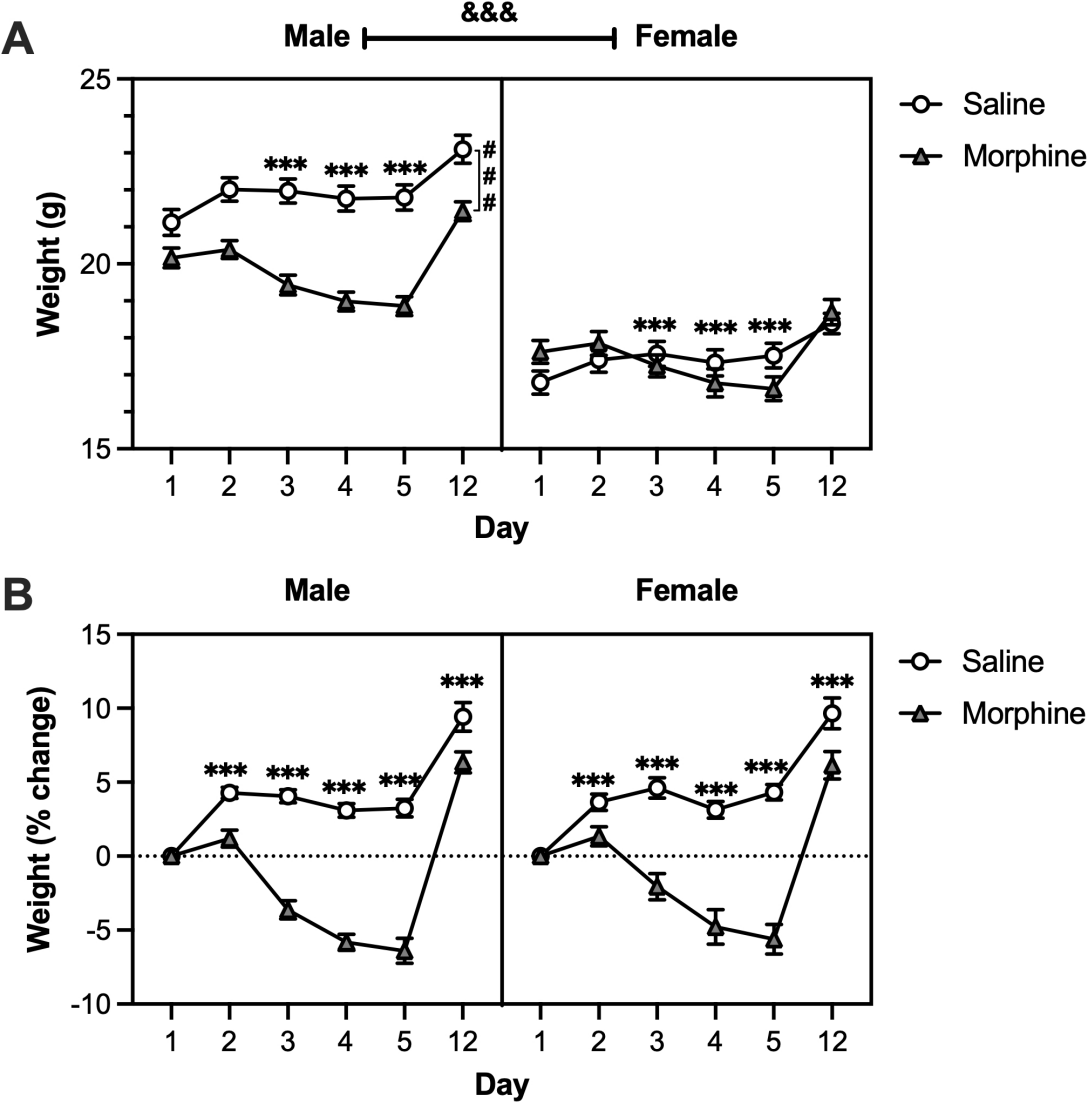
Adolescent morphine exposure blunts weight gain. Adolescent mice received bi-daily escalating doses of morphine (10-50 mg/kg) or saline for 5 days. Body weight was measured each day of drug treatment (Days 1-5) and one week after the final drug administration (Day 12). **A)** Analysis of raw weights revealed that, when averaged across sexes, morphine-treated mice weighed less than saline-treated mice on days 3-5 of treatment (***p < 0.001). When averaged across time, morphine-treated mice weighed less than saline-treated mice within males (^###^p < 0.001), but not females. Overall, females weighed less than males (^&&&^p < 0.001). **B)** Analysis of relative weight change revealed that morphine-treated mice exhibited greater weight loss from baseline than saline-treated mice on days 2-12 of treatment (***p < 0.001). Saline, *n* = 18 males, 16 females; Morphine, *n* = 15 males, 16 females. Data were analyzed using a linear mixed-effects model followed by Bonferroni’s post-hoc tests. Error bars represent ± standard error of mean (SEM).

Adolescent male and female mice were administered bi-daily escalating doses of morphine over five days to induce morphine dependence. To assess morphine-induced changes in body weight, mice were weighed daily during the five days of treatment, and again 1 week after the last injection (Day 12). When examining the raw body weights, a linear mixed-effects model revealed significant main effects of sex [F(1, 61) = 131.3515, p < 0.0001], morphine [F(1, 61) = 12.4615, p < 0.0001], and time [F(5, 305) = 182.746, p < 0.0001]. We also observed significant sex * morphine [F(1, 61) = 11.7574, p = 0.0011, sex * time [F(5, 305) = 3.6453, p = 0.0032, and morphine * time [F(5, 305) = 57.0696, p < 0.0001] interactions. Post-hoc analyses revealed that female mice weighed less than males at all time points (p < 0.0001). When collapsed across time, morphine reduced body weight only in males (p < 0.0001). However, when averaged across sexes, morphine-treated mice weighed significantly less than saline-treated mice on days 3–5 of treatment (p < 0.001) (Fig 2A). Thus, while the non-significant three-way interaction indicates that the temporal effects of morphine do not differ between sexes, the overall morphine * time interaction is likely driven by a larger change in raw weight in males.

To control for baseline body weight differences, raw weights were normalized to percent change from baseline. A linear mixed-effects model revealed a significant main effect of morphine treatment [F(1, 61) = 116.834, p < 0.0001] and time [F(5, 305) = 174.6488, p < 0.0001], in addition to a significant morphine treatment * time interaction [F(5, 305) = 53.9646, p < 0.0001]. Post-hoc analyses revealed that morphine-treated mice exhibited greater weight loss from baseline than saline-treated mice from days 2–12 of treatment (p < 0.001), indicating that morphine’s effect on body weight persists up to 1 week after treatment (Fig 2B).

### LPS treatment reduces body weight and surface temperature

**Fig 3:**
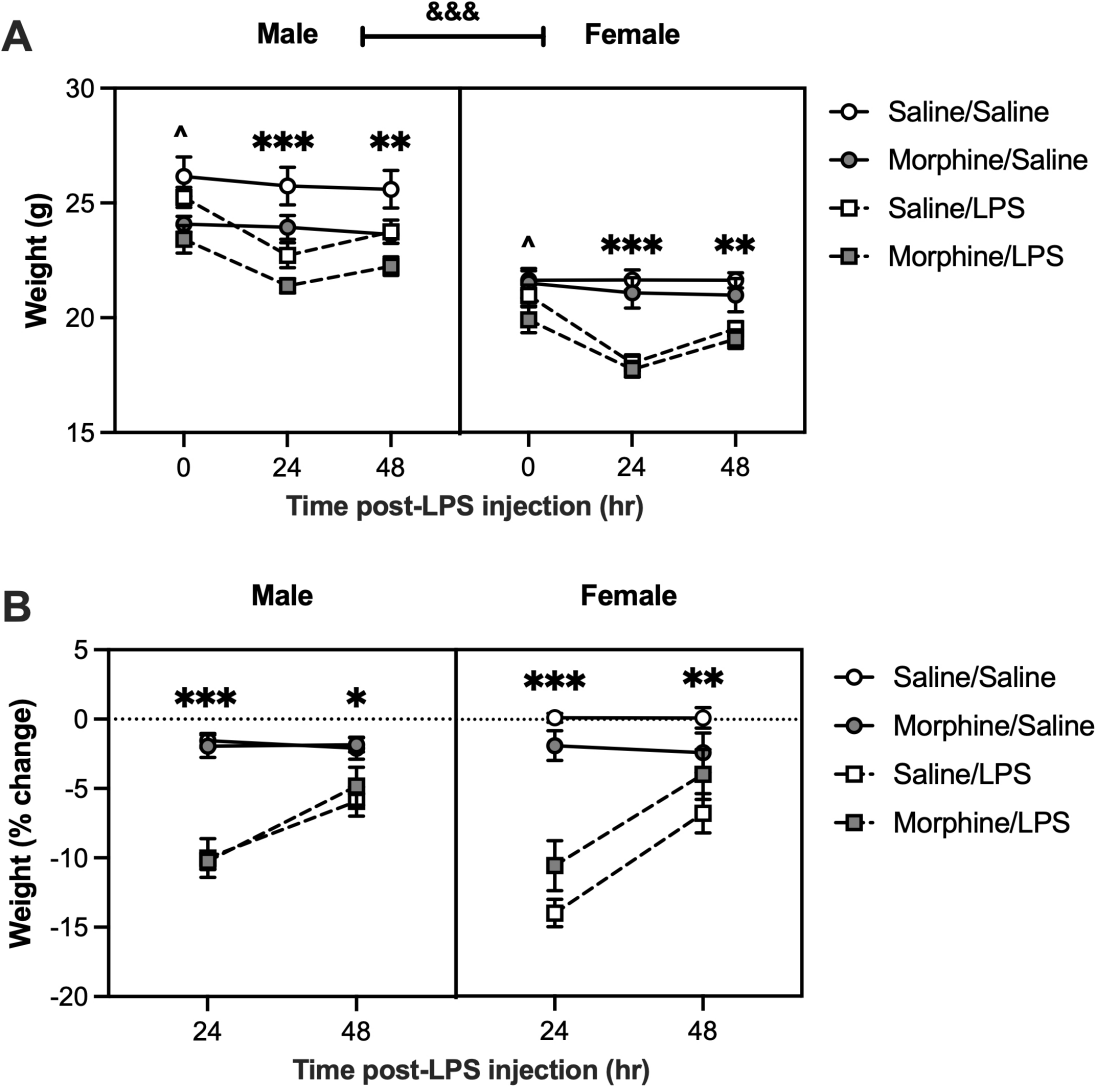
LPS administration reduces body weight. Mice exposed to either saline or morphine during adolescence were later administered LPS (0.1 mg/kg) or saline in adulthood. Body weight was recorded at baseline and then measured 24- and 48-hours post-injection. **A)** Analysis of raw weights revealed that, at baseline, morphine/LPS mice weighed less than saline/LPS mice (^^^p < 0.05). However, in both saline- and morphine-exposed mice, LPS treatment significantly reduced body weight compared to saline treatment at 24 (***p < 0.001) and 48 hours (*p < 0.01) after administration. Overall, females weighed less than males (^&&&^p < 0.001). **B)** Analysis of relative weight change revealed that LPS-treated mice showed greater weight loss from baseline compared to saline-treated mice at 24 hours (***p < 0.001) and 48 hours (*p < 0.05, **p < 0.01) post-injection. Saline/Saline, *n* = 7 males, 8 females; Saline/LPS, *n* = 8 males, 8 females; Morphine/Saline, *n* = 10 males, 8 females; Morphine/LPS, *n* = 8 males, 8 females. Data were analyzed using a linear mixed-effects model followed by Bonferroni’s post-hoc tests. Error bars represent mean ± standard error of mean (SEM).

To determine the effects of LPS treatment on body weight, mice were weighed 1, 24, and 48 hours after LPS administration. Raw weights were analyzed to identify potential baseline differences between mice exposed to saline versus morphine during adolescence. A linear mixed-effects model revealed significant main effects of sex [F(1, 57.023) = 110.4813, p < 0.0001], morphine treatment [F(1, 57.023) = 11.1719, p = 0.0015], LPS treatment [F(1, 57.023) = 31.0753, p < 0.0001], and time [F(2, 113.063) = 122.835, p < 0.0001], in addition to significant interactions of LPS treatment * time [F(2, 113.063) = 71.9136, p < 0.0001] and morphine treatment * LPS treatment * time [F(2, 113.063) = 3.5242, p = 0.0328]. Overall, female mice weighed less than male mice. Post-hoc analyses revealed that at baseline (0 hours), morphine/LPS mice weighed less than saline/LPS mice (p = 0.0382) (Fig 3A), with no other comparisons reaching statistical significance. At 24 hours, both saline-exposed (p < 0.0001) and morphine-exposed (p < 0.0001) mice showed significant LPS-induced weight loss. Similarly, at 48 hours LPS treatment reduced weight in saline-exposed (p = 0.0020) and morphine-exposed (p = 0.0093) mice.

When analyzing relative weight change, a linear mixed-effects model revealed a significant main effect of LPS treatment [F(1,55.957) = 75.327, p < 0.0001], and time [F(1, 54.304) = 115.4889, p < 0.0001], in addition to significant interactions of LPS treatment * time [F(1,54.304) = 138.8027, p < 0.0001], sex * time [F(1,54.304) = 7.0971, p = 0.0101], and sex * LPS treatment * time [F(1,54.304) = 7.2436, p = 0.0094]. Post-hoc analyses indicated that in males, LPS-treated mice had greater weight loss from baseline than saline-treated mice at 24 hours (p < 0.0001) and 48 hours (p = 0.0158) post-injection (Fig 3B). A similar pattern emerged in females, with LPS-treated mice experiencing a greater change from baseline compared to saline-treated mice 24 hours (p < 0.0001) and 48 hours (p = 0.0020) after administration.

A significant morphine treatment * LPS treatment interaction was also observed [F(1,55.957) = 4.1938, p = 0.0453]. Although post-hoc tests failed to identify significant group differences, at 24 hours, the mean percent change from baseline was −10.424 ± 0.972 in morphine/LPS mice compared to −12.003 ± 0.974 in saline/LPS mice, and at 48 hours, −4.414 ± 1.099 versus −6.371 ± 0.862, respectively. Descriptively, these means suggest that morphine-exposed mice experienced less weight loss following LPS administration compared to saline-exposed mice. Nonetheless, the lack of statistically significant pairwise differences likely reflects a subtle effect.

**Fig 4:**
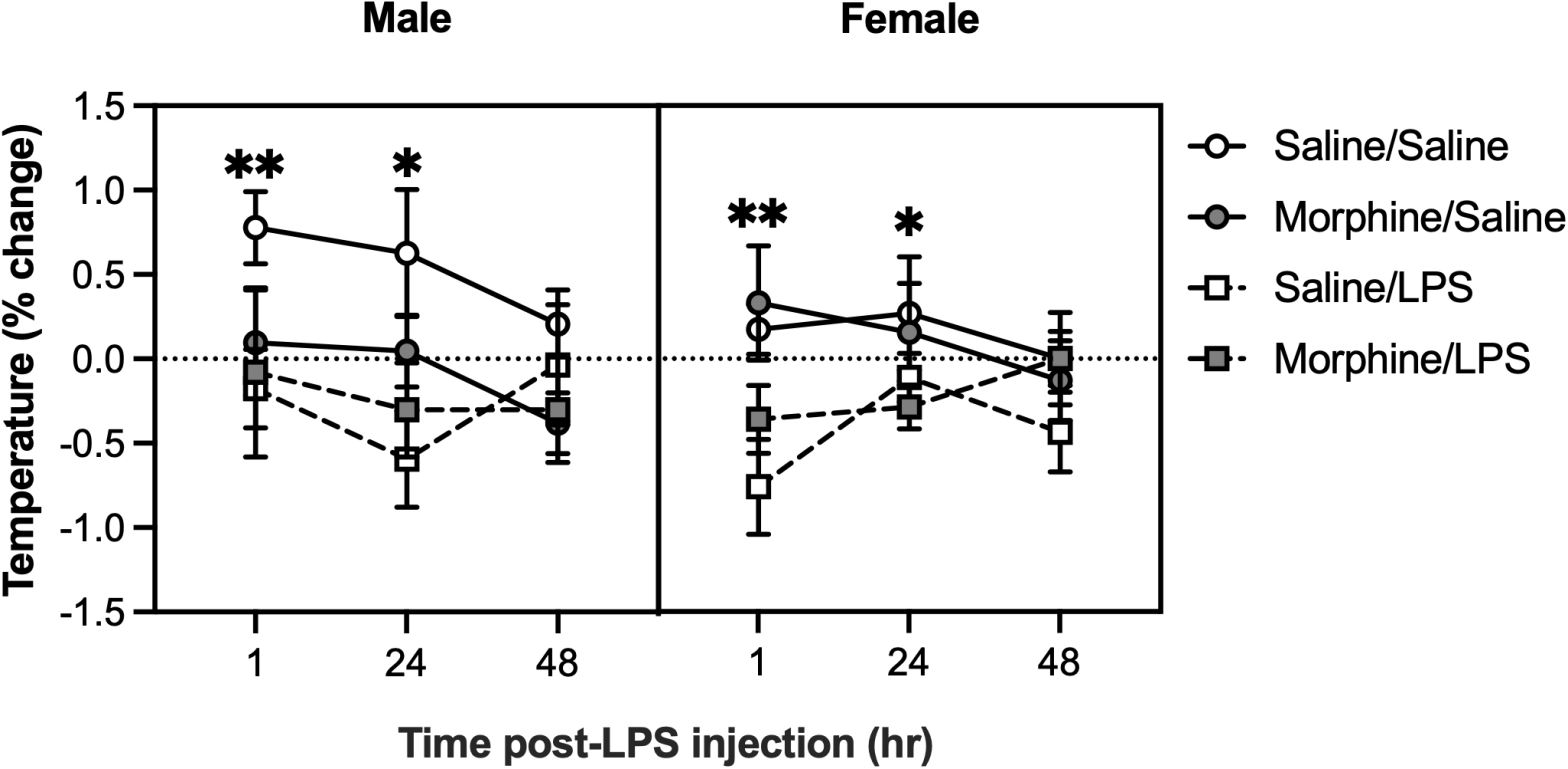
LPS administration reduces abdominal surface temperature. Abdominal surface temperatures were measured at baseline, then 1, 24, and 48 hours post-LPS (0.1 mg/kg) injection. LPS-treated mice exhibited significantly reduced abdominal surface temperature from baseline compared to saline-treated mice 1 hour (**p < 0.01) and 24 hours (*P < 0.05) post-injection. Saline/Saline, *n* = 7 males, 8 females; Saline/LPS, *n* = 8 males, 8 females; Morphine/Saline, *n* = 10 males, 8 females; Morphine/LPS, *n* = 8 males, 8 females. Data were analyzed using a linear mixed-effects model followed by Bonferroni’s post-hoc tests. Error bars represent mean ± standard error of mean (SEM).

Body surface temperature was measured 1, 24, and 48 hours post-LPS injection by aiming an infrared thermometer at the abdomen. Temperatures were normalized to percent change from baseline. A linear mixed-effects model revealed a significant main effect of LPS treatment [F(1,53.09) = 9.5271, p = 0.0032] and a LPS treatment * time interaction [F(2,106.93) = 3.8794, p = 0.0236]. Post-hoc analyses indicated that body surface temperature was reduced in LPS-treated mice compared to saline-treated mice at 1 hour (p = 0.0019) and 24 hours (p = 0.0103) after injection; however, this difference dissipated by 48 hours (Fig 4).

### LPS treatment reduces locomotor activity independent of morphine exposure

**Fig 5:**
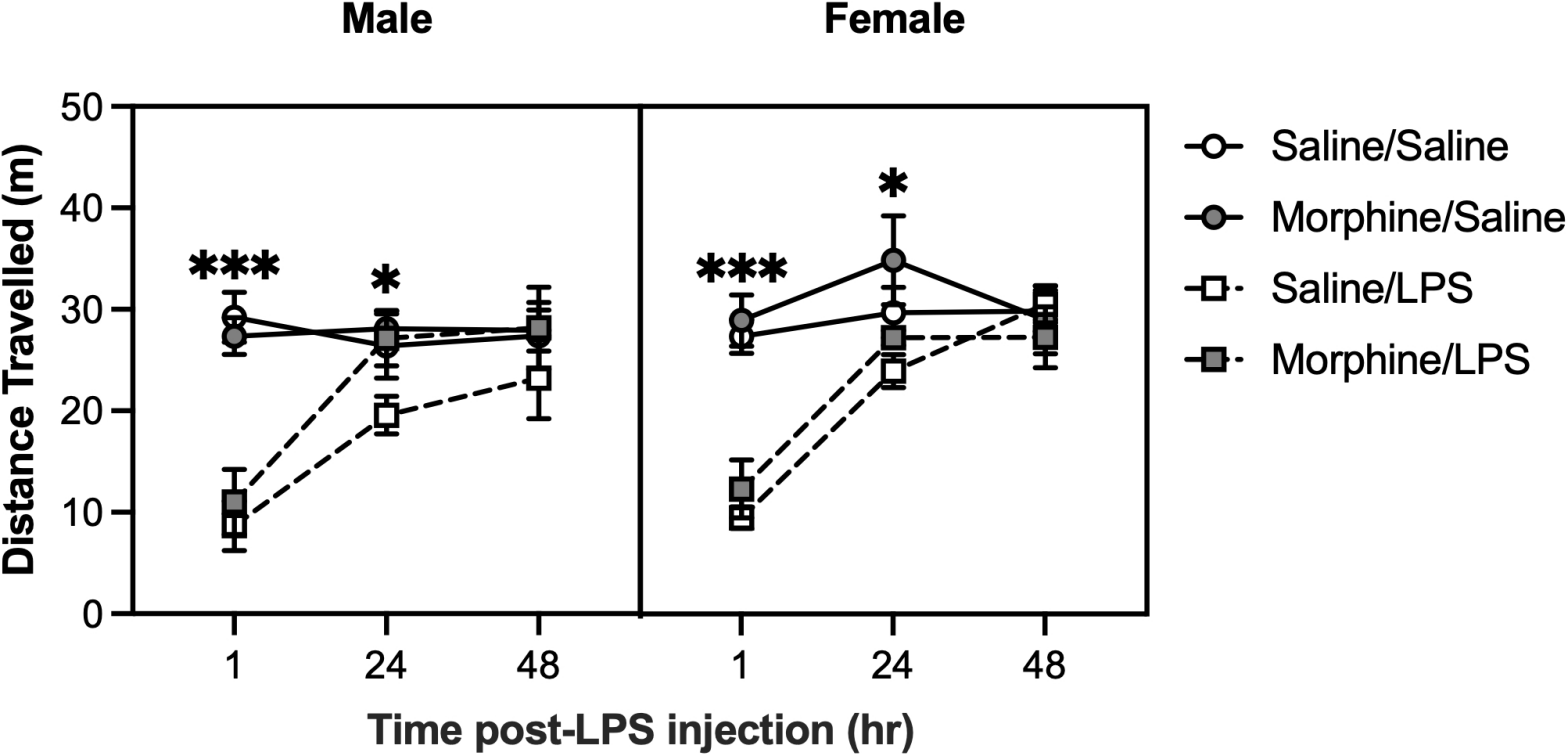
LPS administration reduces locomotor activity. Locomotor activity was assessed 1, 24, and 48 hours after LPS (0.1 mg/kg) or saline administration. Mice treated with LPS travelled less than saline-treated mice when measured 1 hour (***p < 0.001) and 24 hours (p < 0.05) post-injection. Saline/Saline, *n* = 7 males, 8 females; Saline/LPS, *n* = 8 males, 8 females; Morphine/Saline, *n* = 10 males, 8 females; Morphine/LPS, *n* = 8 males, 8 females. Data were analyzed using a mixed-effects model followed by Bonferroni’s post-hoc tests. Error bars represent mean ± standard error of mean (SEM).

Mice were assessed for alterations in locomotor activity 1, 24, and 48 hours after LPS administration. A linear mixed-effect analysis revealed a significant main effect of LPS treatment [F(1,56.532) = 25.0619, p < 0.0001], time [F(2,111.097) = 56.7323, p < 0.0001], and a LPS treatment * time interaction [F(2,111.097) = 47.9985, p < 0.0001]. Post-hoc analyses revealed that compared to saline treatment, LPS treatment reduced locomotor activity 1 hour (p < 0.0001) and 24 hours (p = 0.0154) post-administration (Fig 5). Additionally, there was a significant morphine * time interaction [F(2,111.10) = 3.27, p = 0.0415]; however, post-hoc analyses did not detect any statistically significant differences in locomotor activity between morphine and saline-exposed mice when measured at 1, 24, or 48 hours after saline or LPS administration.

### No effect of drug treatment on behavior in the forced swim test

**Fig 6:**
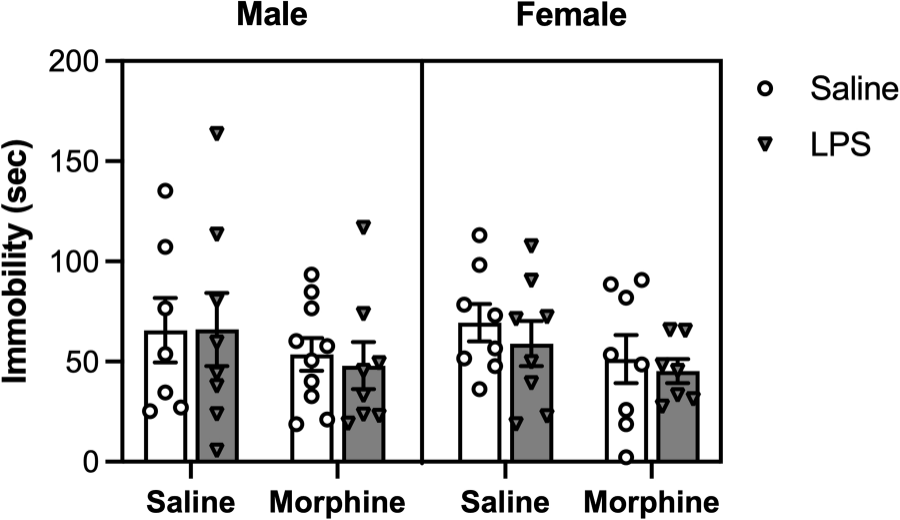
No effect of drug treatment on the forced swim test. Mice were assessed in the forced swim test 52 hours post-LPS administration. There were no differences in time spent immobile across treatment groups or sex. Saline/Saline, *n* = 7 males, 8 females; Saline/LPS, *n* = 8 males, 8 females; Morphine/Saline, *n* = 10 males, 7 females; Morphine/LPS, *n* = 8 males, 8 females. Data were analyzed using a linear model. Error bars represent mean ± standard error of mean (SEM).

The forced swim test was used to measure the effect of adolescent morphine exposure and adult LPS administration on depressive-like behavior. At 52 hours post-LPS injection, mice were assessed for time spent immobile. A linear model analysis indicated no significant effects of morphine treatment [F(1, 56) = 3.1807, p = 0.07993], LPS treatment [F(1, 56) = 0.1019, p = 0.75073], or sex [F(1, 56) = 0.021, p = 0.88541] (Fig 6).

## Discussion

There is mounting clinical and preclinical evidence indicating that early-life opioid exposure can alter immune activation later in life [45,48,49,53]. The goal of the present study was to determine whether adolescent morphine exposure in mice alters sickness behaviors in response to an immune challenge in adulthood. Morphine dependence was achieved utilizing an escalating dosing schedule previously shown to produce robust autonomic and somatic withdrawal symptoms in adolescent mice [56]. Weight loss is a well-documented marker of morphine-induced metabolic changes in rodents [63,64], and adolescent morphine exposure has been demonstrated to have a long-lasting impact on body weight [65]. Consistent with these findings, we found that mice in the adolescent morphine treatment group showed a greater reduction in weight compared to saline controls. Morphine had more of an impact on raw weight in males, which may reflect increased sensitivity to the somatic effects of opioids [66,67]. Nonetheless, both males and females treated with morphine experienced significant weight loss from baseline beginning on the second day of drug administration and lasting up to at least a week after the cessation of treatment. This suggests that the morphine dose and administration paradigm utilized in this study effectively produced physiological changes in both sexes.

Findings from both the clinical and preclinical literature indicate that early-life opioid exposure sensitizes the immune system [44,45,48,49], which may contribute to increased immune vulnerability later in life. Lipopolysaccharide (LPS), an endotoxin located on the outer membrane of gram-negative bacteria, is a commonly used tool to investigate immune response in both humans and animal models due to its ability to activate the innate immune system and induce many symptoms of sickness, including weight loss, lethargy, and alterations in mood, among others [28,29]. Whereas high doses of LPS (≥ 5 mg/kg) can trigger sepsis and severe sickness symptoms in rodents, lower doses (≤ 1 mg/kg) produce a submaximal immune response that more closely mimics a mild infection [68].

Previous investigations indicate that prior insults can exacerbate the physiological and behavioral effects of a low-dose LPS challenge [58,69]. Given this, we predicted that mice with a history of adolescent morphine exposure would exhibit a potentiated sickness response to a low-dose LPS (0.1 mg/kg) challenge. Contrary to our hypotheses, LPS-treated mice presented with similar sickness symptoms regardless of adolescent drug exposure. LPS treatment suppressed locomotor activity for 24 hours and reduced body weight for up to 48 hours in both saline- and morphine-exposed mice. Notably, morphine/LPS mice weighed less than saline/LPS mice at baseline (before injection). However, seeing as mice were randomly assigned to LPS or saline treatment, this finding likely reflects a general deleterious effect of adolescent morphine exposure on adult body weight. This aligns with existing rodent literature demonstrating that adolescent morphine exposure reduces weight gain for up to four weeks [65].

Our analysis of weight change from baseline revealed a significant interaction between adolescent morphine exposure and LPS treatment. Although follow-up pairwise comparisons were not significant, group means suggest that LPS-induced weight loss was less severe in morphine-exposed mice compared to their saline-exposed counterparts. While contrary to our initial hypotheses, there is evidence that immune activation during critical developmental windows can attenuate responses to later immune challenges [70]. Nonetheless, the lack of significant pairwise differences likely reflects subtle effects of adolescent morphine exposure on LPS-induced weight changes and should be interpreted with caution.

Interestingly, LPS treatment reduced body surface temperature for up to 24 hours, in contrast to the febrile response often associated with systemic inflammation [71]. This could be due to surface-level vasoconstriction, a known response to LPS in mouse tail skin that coincides with a rise in core body temperature [72]. Alternatively, this finding may indicate a hypothermic core temperature response to LPS, which has previously been reported in mice [60,73] and may be a protective mechanism against LPS-induced shock [74]. In the present study, body temperature was measured using an infrared thermometer, which is considered to be a reliable non-invasive method for assessing body temperature in mice [59,60]. However, while potentially more distressing to animals, measuring core temperature via a rectal thermometer or implantable probe may have uncovered interactions between adolescent opioid exposure and adult sickness symptoms. Indeed, data collected from temperature sensors implanted in the abdominal cavity revealed that perinatal morphine exposure potentiated LPS-induced fever in adult female rats [49]. Furthermore, since implantable thermometers can collect more frequent data points over an extended period, this method may be better suited for detecting subtle or temporal effects.

Chronic neuroinflammation is associated with anxiety and depression-like symptoms in rodents [34–36]. A septic dose of LPS (5 mg/kg) increases anxiety and behavioral despair in mice for up to a month after treatment [75]. Lower doses of LPS (0.1–0.83 mg/kg) also produce anxiety- and depressive-like behavior; however, these effects are typically observed within 24 hours of administration [76–78]. Notably, Couch and colleagues found that prior exposure to chronic stress augmented the effects of 0.1 mg/kg LPS on immobility in the forced swim test (FST) 24 hours after administration [58]. The authors did not note locomotor deficits in LPS-treated mice at this time point; however, other studies using low-dose LPS have demonstrated reduced motor activity at 24 hours [41,79]. To avoid the confound of motor deficits impacting swim behavior in the FST, we conducted the assay 52 hours after LPS administration to confirm normal locomotor function in LPS-treated mice. We did not detect any differences in depressive-like behavior among treatment groups; however, any subtle or transient depressive-like effects of LPS treatment may have dissipated by this time point. Alternatively, a larger dose of LPS may be necessary to identify persistent alterations in behavior. More work is needed to fully characterize the temporal profile of LPS-induced affective behavior in this model.

Recent studies have identified changes in neuroimmune-related adult behavior following adolescent morphine exposure, such as increased inflammatory pain [80]. In these studies, morphine treatment was initiated during a period commonly defined as early adolescence in rodents (postnatal day 21–34), whereas mice in the current study began treatment during mid-adolescence, which is defined as postnatal day 34–46 [81–83]. Neurobehavioral sensitivity to psychoactive drugs differs between stages of adolescence [82,84]; thus, earlier developmental periods may be more susceptible to morphine-mediated effects on the immune system. Additionally, since females tend to enter puberty earlier than males [85–88], future experiments should consider sex-specific critical periods within adolescence, in addition to sex differences in immune activity [89,90]. For example, females have been shown to have stronger immune responses but recover faster from immune challenges compared to males; an effect thought to be mediated by estrogen [91].

A limitation of the current study is that only one dose of LPS was investigated. LPS dose-dependently initiates the production of pro-inflammatory cytokines [30,32,92]. It is possible that the dose of LPS used in the current study was sufficient to generate some sickness behaviors without promoting a full immune response. Future studies could examine higher doses of LPS to investigate the impact of adolescent morphine exposure in more severe inflammatory states, like sepsis. Implementing high-speed videography and machine learning approaches in future research would facilitate objective measurement of physical discomfort in rodents, such as piloerection, ptosis, ear changes, orbital tightening, and nose/cheek flattening [93,94]. The current study was also limited by its focus on a narrow window of adolescent morphine exposure and adult testing, which prevented us from identifying time-specific effects. Lastly, the scope of the current study was limited to behavioral and physiological measurements; thus, it is unclear whether adolescent morphine exposure altered neuroimmune response to LPS on a molecular level. Future research may seek to quantify microglial activation, the presence of peripheral macrophages in the brain, and the expression of pro- and anti-inflammatory cytokines to assess short-term and long-term neuroinflammatory states following LPS exposure.

Further investigation is needed to fully elucidate the long-term neuroimmune effects of early-life opioid exposure. Greater insight into how opioids affect immune system signaling during sensitive developmental periods is important for enhancing health outcomes in vulnerable populations impacted by the opioid crisis.

## Supporting information

S1 Table Statistics table for Fig 2. Drug = saline or morphine.
(DOCX)

S2 Table Statistics table for Fig 3. Drug1 = saline or morphine. Drug2 = saline or lipopolysaccharide (LPS).
(DOCX)

S3 Table Statistics table for Fig 4. Drug1 = saline or morphine. Drug2 = saline or lipopolysaccharide (LPS).
(DOCX)

S4 Table Statistics table for Fig 5. Drug1 = saline or morphine. Drug2 = saline or lipopolysaccharide (LPS).
(DOCX)

S5 Table Statistics table for Fig 6. Drug1 = saline or morphine. Drug2 = saline or lipopolysaccharide (LPS).
(DOCX)
