# Supplementary material for: Adolescent morphine exposure does not alter low-dose lipopolysaccharide (LPS)-induced sickness behavior in adult C57/BL6 mice
Source: PLoS One. 2025 Nov 4;20(11):e0328026. doi: 10.1371/journal.pone.0328026 (PMC12585049; doi:10.1371/journal.pone.0328026)
Supplement: S1 Table — Drug = saline or morphine. (DOCX) [file pone.0328026.s001.docx]

| **Fig 2A: Adolescent morphine exposure blunts weight gain - Analysis of raw weights** | | | | | |  |
| --- | --- | --- | --- | --- | --- | --- |
| **Effect** | **Sum Sq** | **Mean Sq** | **NumDF** | **DenDF** | **F value** | **p value** |
| Sex | 19.296 | 19.2956 | 1 | 61 | 131.3515 | < 2.2E-16 |
| Drug | 1.831 | 1.8306 | 1 | 61 | 12.4615 | 7.97E-04 |
| Time | 134.227 | 26.8455 | 5 | 305 | 182.746 | < 2.2E-16 |
| Sex:Drug | 1.727 | 1.7272 | 1 | 61 | 11.7574 | 0.0011 |
| Sex:Time | 2.677 | 0.5355 | 5 | 305 | 3.6453 | 0.0032 |
| Drug:Time | 41.918 | 8.3835 | 5 | 305 | 57.0696 | < 2.2E-16 |
| Sex:Drug:Time | 0.561 | 0.1121 | 5 | 305 | 0.7632 | 0.5769 |
| **Drug:Time** | **Contrast** | **Estimate** | **SE** | **df** | **t ratio** | **p value** |
| Day 1 | Morphine-Saline | -0.0659 | 0.312 | 71.6 | -0.211 | 1 |
| Day 2 | Morphine-Saline | -0.587 | 0.312 | 71.6 | -1.882 | 0.3836 |
| Day 3 | Morphine-Saline | -1.4328 | 0.312 | 71.6 | -4.593 | 0.0001 |
| Day 4 | Morphine-Saline | -1.6636 | 0.312 | 71.6 | -5.333 | <.0001 |
| Day 5 | Morphine-Saline | -1.9153 | 0.312 | 71.6 | -6.14 | <.0001 |
| Day 12 | Morphine-Saline | -0.6807 | 0.312 | 71.6 | -2.182 | 0.1943 |
| **Sex: Time** | **Contrast** | **Estimate** | **SE** | **df** | **t ratio** | **p value** |
| Day 1 | Female-Male | -3.44 | 0.312 | 71.6 | -11.013 | <.0001 |
| Day 2 | Female-Male | -3.57 | 0.312 | 71.6 | -11.454 | <.0001 |
| Day 3 | Female-Male | -3.29 | 0.312 | 71.6 | -10.553 | <.0001 |
| Day 4 | Female-Male | -3.32 | 0.312 | 71.6 | -10.646 | <.0001 |
| Day 5 | Female-Male | -3.26 | 0.312 | 71.6 | -10.445 | <.0001 |
| Day 12 | Female-Male | -3.72 | 0.312 | 71.6 | -11.928 | <.0001 |
| **Sex: Drug** | **Contrast** | **Estimate** | **SE** | **df** | **t ratio** | **p value** |
| Female | Morphine-Saline | -0.0303 | 0.426 | 61 | -0.071 | 1 |
| Male | Morphine-Saline | -2.0848 | 0.421 | 61 | -4.949 | <.0001 |
| **Fig 2B: Adolescent morphine exposure blunts weight gain - Analysis of relative weight change** | | | | | | |
| **Effect** | **Sum Sq** | **Mean Sq** | **NumDF** | **DenDF** | **F value** | **p value** |
| Sex | 2.8 | 2.78 | 1 | 61 | 0.6413 | 0.4263 |
| Drug | 506.2 | 506.19 | 1 | 61 | 116.834 | 8.36E-16 |
| Time | 3783.4 | 756.67 | 5 | 305 | 174.6488 | < 2.2E-16 |
| Sex:Drug | 0.5 | 0.54 | 1 | 61 | 0.125 | 0.7249 |
| Sex:Time | 23.5 | 4.71 | 5 | 305 | 1.0863 | 0.3679 |
| Drug:Time | 1169 | 233.8 | 5 | 305 | 53.9646 | < 2.2E-16 |
| Sex:Drug:Time | 8.9 | 1.78 | 5 | 305 | 0.4097 | 0.8420 |
| **Drug:Time** | **Contrast** | **Estimate** | **SE** | **df** | **t ratio** | **p value** |
| Day 1 | Morphine-Saline | 0 | 0.676 | 197 | 0 | 1 |
| Day 2 | Morphine-Saline | -2.69 | 0.676 | 197 | -3.984 | 0.0006 |
| Day 3 | Morphine-Saline | -7.19 | 0.676 | 197 | -10.626 | <.0001 |
| Day 4 | Morphine-Saline | -8.42 | 0.676 | 197 | -12.449 | <.0001 |
| Day 5 | Morphine-Saline | -9.78 | 0.676 | 197 | -14.47 | <.0001 |
| Day 12 | Morphine-Saline | -3.3 | 0.676 | 197 | -4.878 | <.0001 |

Drug = saline or morphine.
