# Supplementary material for: Adolescent morphine exposure does not alter low-dose lipopolysaccharide (LPS)-induced sickness behavior in adult C57/BL6 mice
Source: PLoS One. 2025 Nov 4;20(11):e0328026. doi: 10.1371/journal.pone.0328026 (PMC12585049; doi:10.1371/journal.pone.0328026)
Supplement: S2 Table — Drug1 = saline or morphine. Drug2 = saline or lipopolysaccharide (LPS). (DOCX) [file pone.0328026.s002.docx]

| **Fig 3A: LPS administration reduces body weight - Analysis of raw weights** | | | | |  |  |
| --- | --- | --- | --- | --- | --- | --- |
| **Effect** | **Sum Sq** | **Mean Sq** | **NumDF** | **DenDF** | **F value** | **p value** |
| Sex | 27.155 | 27.155 | 1 | 57.023 | 110.4813 | 5.83E-15 |
| Drug1 | 2.746 | 2.7459 | 1 | 57.023 | 11.1719 | 0.0015 |
| Drug2 | 7.638 | 7.6379 | 1 | 57.023 | 31.0753 | 7.07E-07 |
| Time | 60.383 | 30.1914 | 2 | 113.063 | 122.835 | < 2.2E-16 |
| Sex:Drug1 | 0.835 | 0.8349 | 1 | 57.023 | 3.3966 | 0.0705 |
| Sex:Drug2 | 0.145 | 0.1452 | 1 | 57.023 | 0.5908 | 0.4453 |
| Drug1:Drug2 | 0.015 | 0.0152 | 1 | 57.023 | 0.062 | 0.8043 |
| Sex:Time | 0.535 | 0.2676 | 2 | 113.063 | 1.0886 | 0.3402 |
| Drug1:Time | 0.331 | 0.1656 | 2 | 113.063 | 0.6738 | 0.5118 |
| Drug2:Time | 35.351 | 17.6755 | 2 | 113.063 | 71.9136 | < 2.2E-16 |
| Sex:Drug1:Drug2 | 0.055 | 0.0551 | 1 | 57.023 | 0.2241 | 0.6377 |
| Sex:Drug1:Time | 0.063 | 0.0314 | 2 | 113.063 | 0.1279 | 0.8800 |
| Sex:Drug2:Time | 0.657 | 0.3283 | 2 | 113.063 | 1.3356 | 0.2671 |
| Drug1:Drug2:Time | 1.732 | 0.8662 | 2 | 113.063 | 3.5242 | 0.0328 |
| Sex:Drug1:Drug2:Time | 0.463 | 0.2314 | 2 | 113.063 | 0.9414 | 0.3931 |
| **Drug2:Time** | **Contrast** | **Estimate** | **SE** | **df** | **t ratio** | **p value** |
| 0 hours | LPS-Saline | -0.955 | 0.362 | 66.7 | -2.635 | 0.0314 |
| 24 hours | LPS-Saline | -3.048 | 0.363 | 66.9 | -8.405 | <.0001 |
| 48 hours | LPS-Saline | -1.818 | 0.362 | 66.7 | -5.017 | <.0001 |
| **Drug1:Drug2:Time** | **Contrast** | **Estimate** | **SE** | **df** | **t ratio** | **p value** |
| LPS_0 hours | Morphine-Saline | -1.449 | 0.514 | 66.7 | -2.817 | 0.0382 |
| Saline_0 hours | Morphine-Saline | -1.1 | 0.511 | 66.7 | -2.154 | 0.2093 |
| LPS_24 hours | Morphine-Saline | -0.804 | 0.514 | 66.7 | -1.563 | 0.7367 |
| Saline_24 hours | Morphine-Saline | -1.345 | 0.512 | 67.2 | -2.629 | 0.0636 |
| LPS_ 48 hours | Morphine-Saline | -0.978 | 0.514 | 66.7 | -1.901 | 0.3699 |
| Saline_48 hours | Morphine-Saline | -1.305 | 0.511 | 66.7 | -2.557 | 0.0771 |
| **Drug1:Drug2:Time** | **Contrast** | **Estimate** | **SE** | **df** | **t ratio** | **p value** |
| Morphine_ 0 hours | LPS - Saline | -1.13 | 0.501 | 66.7 | -2.253 | 0.1653 |
| Saline_ 0 hours | LPS - Saline | -0.78 | 0.523 | 66.7 | -1.491 | 0.8447 |
| Morphine_24 hours | LPS - Saline | -2.78 | 0.502 | 67.2 | -5.53 | <.0001 |
| Saline_ 24 hours | LPS - Saline | -3.32 | 0.523 | 66.7 | -6.341 | <.0001 |
| Morphine_48 hours | LPS - Saline | -1.65 | 0.501 | 66.7 | -3.3 | 0.0093 |
| Saline_48 hours | LPS - Saline | -1.98 | 0.523 | 66.7 | -3.787 | 0.0020 |
| **Fig 3B: LPS administration reduces body weight - Analysis of relative weight change** | | | | | |  |
| **Effect** | **Sum Sq** | **Mean Sq** | **NumDF** | **DenDF** | **F value** | **p value** |
| Sex | 0.234 | 0.234 | 1 | 55.957 | 0.1219 | 0.7283 |
| Drug1 | 0.601 | 0.601 | 1 | 55.957 | 0.3135 | 0.5778 |
| Drug2 | 144.431 | 144.431 | 1 | 55.957 | 75.327 | 6.02E-12 |
| Time | 221.437 | 221.437 | 1 | 54.304 | 115.4889 | 4.68E-15 |
| Sex:Drug1 | 0 | 0 | 1 | 55.957 | 0.0001 | 0.9930 |
| Sex:Drug2 | 7.28 | 7.28 | 1 | 55.957 | 1.7442 | 0.1920 |
| Drug1:Drug2 | 8.041 | 8.041 | 1 | 55.957 | 4.1938 | 0.0453 |
| Sex:Time | 13.608 | 13.608 | 1 | 54.304 | 7.0971 | 0.0101 |
| Drug1:Time | 0.018 | 0.018 | 1 | 54.304 | 0.0095 | 0.9227 |
| Drug2:Time | 266.139 | 266.139 | 1 | 54.304 | 138.8027 | < 2.2E-16 |
| Sex:Drug1:Drug2 | 3.849 | 3.849 | 1 | 55.957 | 2.0076 | 0.1621 |
| Sex:Drug1:Time | 2.051 | 2.051 | 1 | 54.304 | 1.0697 | 0.3056 |
| Sex:Drug2:Time | 13.889 | 13.889 | 1 | 54.304 | 7.2436 | 0.0094 |
| Drug1:Drug2:Time | 0.052 | 0.052 | 1 | 54.304 | 0.027 | 0.8701 |
| Sex:Drug1:Drug2:Time | 0.007 | 0.007 | 1 | 54.304 | 0.0035 | 0.9529 |
| **Drug1:Drug2** | **Contrast** | **Estimate** | **SE** | **df** | **t ratio** | **p value** |
| LPS | Morphine - Saline | 2.02 | 1.1 | 57 | 1.837 | 0.1429 |
| Saline | Morphine - Saline | -1.15 | 1.09 | 56.9 | -1.056 | 0.5906 |
| **Drug2:Time** | **Contrast** | **Estimate** | **SE** | **df** | **t ratio** | **p value** |
| 24 hrs | LPS - Saline | -9.64 | 0.816 | 68.8 | -11.819 | <.0001 |
| 48 hrs | LPS - Saline | -3.82 | 0.812 | 67.8 | -4.702 | <.0001 |
| **Sex:Time** | **Contrast** | **Estimate** | **SE** | **df** | **t ratio** | **p value** |
| 24 hrs | Female - Male | -0.929 | 0.816 | 68.8 | -1.139 | 0.5173 |
| 48 hrs | Female - Male | 0.388 | 0.812 | 67.8 | 0.478 | 1 |
| **Sex:Drug2:Time** | **Contrast** | **Estimate** | **SE** | **df** | **t ratio** | **p value** |
| LPS_24 hours | Female - Male | -2.6638 | 1.16 | 69.1 | -2.299 | 0.0982 |
| Saline_24 hours | Female - Male | 0.8057 | 1.15 | 68.6 | 0.702 | 1 |
| LPS_48 hours | Female - Male | -0.0169 | 1.15 | 67.8 | -0.015 | 1 |
| Saline_48 hours | Female - Male | 0.7922 | 1.14 | 67.8 | 0.693 | 1 |
| **Sex:Drug2:Time** | **Contrast** | **Estimate** | **SE** | **df** | **t ratio** | **p value** |
| Female_24 hours | LPS - Saline | -11.37 | 1.15 | 67.8 | -9.873 | <.0001 |
| Male_24 hours | LPS - Saline | -7.9 | 1.15 | 69.9 | -6.845 | <.0001 |
| Female_48 hours | LPS - Saline | -4.22 | 1.15 | 67.8 | -3.664 | 0.0020 |
| Male_48 hours | LPS - Saline | -3.41 | 1.14 | 67.8 | -2.983 | 0.0158 |

Drug1 = saline or morphine. Drug2 = saline or lipopolysaccharide (LPS).
