# Supplementary material for: Adolescent morphine exposure does not alter low-dose lipopolysaccharide (LPS)-induced sickness behavior in adult C57/BL6 mice
Source: PLoS One. 2025 Nov 4;20(11):e0328026. doi: 10.1371/journal.pone.0328026 (PMC12585049; doi:10.1371/journal.pone.0328026)
Supplement: S3 Table — Drug1 = saline or morphine. Drug2 = saline or lipopolysaccharide (LPS). (DOCX) [file pone.0328026.s003.docx]

| **Fig 4: LPS administration reduces abdominal surface temperature** | | | | |  |  |
| --- | --- | --- | --- | --- | --- | --- |
| **Effect** | **Sum Sq** | **Mean Sq** | **NumDF** | **DenDF** | **F value** | **p value** |
| Sex | 0.068 | 0.068 | 1 | 53.09 | 0.1774 | 0.6753 |
| Drug1 | 0.0914 | 0.0914 | 1 | 53.09 | 0.2385 | 0.6273 |
| Drug2 | 3.6506 | 3.6506 | 1 | 53.09 | 9.5271 | 0.0032 |
| Time | 0.7342 | 0.3671 | 2 | 106.93 | 0.958 | 0.3869 |
| Sex:Drug1 | 0.4936 | 0.4936 | 1 | 53.09 | 1.2883 | 0.2614 |
| Sex:Drug2 | 0 | 0 | 1 | 53.09 | 0.0001 | 0.9930 |
| Drug1:Drug2 | 0.8183 | 0.8183 | 1 | 53.09 | 2.1355 | 0.1498 |
| Sex:Time | 0.8886 | 0.4443 | 2 | 106.93 | 1.1595 | 0.3176 |
| Drug1:Time | 0.3363 | 0.1681 | 2 | 106.93 | 0.4388 | 0.6460 |
| Drug2:Time | 2.9731 | 1.4865 | 2 | 106.93 | 3.8794 | 0.0236 |
| Sex:Drug1:Drug2 | 0.1477 | 0.1477 | 1 | 53.09 | 0.3853 | 0.5374 |
| Sex:Drug1:Time | 0.5242 | 0.2621 | 2 | 106.93 | 0.684 | 0.5068 |
| Sex:Drug2:Time | 0.7229 | 0.3615 | 2 | 106.93 | 0.9433 | 0.3925 |
| Drug1:Drug2:Time | 0.0389 | 0.0195 | 2 | 106.93 | 0.0508 | 0.9505 |
| Sex:Drug1:Drug2:Time | 0.6903 | 0.3452 | 2 | 106.93 | 0.9008 | 0.4093 |
| **Drug2:Time** | **Contrast** | **Estimate** | **SE** | **df** | **t ratio** | **p value** |
| 1 hour | LPS - Saline | -0.688 | 0.197 | 135 | -3.492 | 0.0019 |
| 24 hours | LPS - Saline | -0.581 | 0.195 | 133 | -2.98 | 0.0103 |
| 48 hours | LPS - Saline | -0.104 | 0.197 | 135 | -0.528 | 1 |

Drug1 = saline or morphine. Drug2 = saline or lipopolysaccharide (LPS).
