# Supplementary material for: Adolescent morphine exposure does not alter low-dose lipopolysaccharide (LPS)-induced sickness behavior in adult C57/BL6 mice
Source: PLoS One. 2025 Nov 4;20(11):e0328026. doi: 10.1371/journal.pone.0328026 (PMC12585049; doi:10.1371/journal.pone.0328026)
Supplement: S4 Table — Drug1 = saline or morphine. Drug2 = saline or lipopolysaccharide (LPS). (DOCX) [file pone.0328026.s004.docx]

| **Fig 5: LPS administration reduces locomotor activity** | | | |  |  |  |
| --- | --- | --- | --- | --- | --- | --- |
| **Effect** | **Sum Sq** | **Mean Sq** | **NumDF** | **DenDF** | **F value** | **p value** |
| Sex | 40.38 | 40.38 | 1 | 56.532 | 1.5991 | 0.2112 |
| Drug1 | 35.21 | 35.21 | 1 | 56.532 | 1.3943 | 0.2426 |
| Drug2 | 632.89 | 632.89 | 1 | 56.532 | 25.0619 | 5.76E-06 |
| Time | 2865.34 | 1432.67 | 2 | 111.097 | 56.7323 | < 2.2E-16 |
| Sex:Drug1 | 0.4 | 0.4 | 1 | 56.532 | 0.0157 | 0.9006 |
| Sex:Drug2 | 0.39 | 0.39 | 1 | 56.532 | 0.0153 | 0.9021 |
| Drug1:Drug2 | 5.62 | 5.62 | 1 | 56.532 | 0.2225 | 0.6390 |
| Sex:Time | 95.59 | 47.8 | 2 | 111.097 | 1.8927 | 0.1555 |
| Drug1:Time | 165.37 | 82.69 | 2 | 111.097 | 3.2742 | 0.0415 |
| Drug2:Time | 2424.23 | 1212.11 | 2 | 111.097 | 47.9985 | 9.46E-16 |
| Sex:Drug1:Drug2 | 14.07 | 14.07 | 1 | 56.532 | 0.5571 | 0.4585 |
| Sex:Drug1:Time | 65.05 | 32.53 | 2 | 111.097 | 1.288 | 0.2799 |
| Sex:Drug2:Time | 35.32 | 17.66 | 2 | 111.097 | 0.6993 | 0.4991 |
| Drug1:Drug2:Time | 8.62 | 4.31 | 2 | 111.097 | 0.1708 | 0.8432 |
| Sex:Drug1:Drug2:Time | 17.57 | 8.79 | 2 | 111.097 | 0.3479 | 0.7069 |
| **Drug1: Time** | **Contrast** | **Estimate** | **SE** | **df** | **t ratio** | **p value** |
| 1 hour | Morphine-Saline | 0.937 | 1.87 | 108 | 0.502 | 1 |
| 24 hours | Morphine-Saline | 4.428 | 1.86 | 106 | 2.386 | 0.0564 |
| 48 hours | Morphine-Saline | 0.136 | 1.88 | 109 | 0.072 | 1 |
| **Drug2: Time** | **Contrast** | **Estimate** | **SE** | **df** | **t ratio** | **p value** |
| 1 hour | LPS - Saline | -17.555 | 1.87 | 108 | -9.407 | <.0001 |
| 24 hours | LPS - Saline | -5.303 | 1.86 | 106 | -2.858 | 0.0154 |
| 48 hours | LPS - Saline | -0.465 | 1.88 | 109 | -0.248 | 1 |

Drug1 = saline or morphine. Drug2 = saline or lipopolysaccharide (LPS).
