# Supplementary material for: Adolescent morphine exposure does not alter low-dose lipopolysaccharide (LPS)-induced sickness behavior in adult C57/BL6 mice
Source: PLoS One. 2025 Nov 4;20(11):e0328026. doi: 10.1371/journal.pone.0328026 (PMC12585049; doi:10.1371/journal.pone.0328026)
Supplement: S5 Table — Drug1 = saline or morphine. Drug2 = saline or lipopolysaccharide (LPS). (DOCX) [file pone.0328026.s005.docx]

| **Fig 6: No effect of drug treatment on the forced swim test** | | | |  |  |
| --- | --- | --- | --- | --- | --- |
| **Effect** | **Df** | **Sum Sq** | **Mean Sq** | **F value** | **p value** |
| Sex | 1 | 24 | 24.3 | 0.021 | 0.88541 |
| Drug1 | 1 | 3684 | 3684.3 | 3.1807 | 0.07993 |
| Drug2 | 1 | 118 | 118.1 | 0.1019 | 0.75073 |
| Sex:Drug1 | 1 | 2 | 1.7 | 0.0015 | 0.96970 |
| Sex:Drug2 | 1 | 1 | 0.7 | 0.0006 | 0.98016 |
| Drug1:Drug2 | 1 | 96 | 95.5 | 0.0825 | 0.77506 |
| Sex:Drug1:Drug2 | 1 | 496 | 495.8 | 0.428 | 0.51564 |
| Residuals | 56 | 64866 | 1158.3 |  |  |

Drug1 = saline or morphine. Drug2 = saline or lipopolysaccharide (LPS).
